# Update on new research in Gram-negative infections European Congress of Clinical Microbiology and Infectious Diseases 2017 (22–25 April, Vienna, Austria)

**DOI:** 10.4155/fsoa-2017-0132

**Published:** 2017-11-30

**Authors:** Matteo Bassetti, Susan Mayor

**Affiliations:** 1 Department of Medicine, University of Udine & Santa Maria Misericordia University Hospital, Udine, Italy; 2 London, UK

**Keywords:** antimicrobial therapy, carbapenamase-producing *Enterobacteriaceae*, Gram-negative, *Pseudomonas aeruginosa*

## Abstract

European Congress of Clinical Microbiology and Infectious Diseases 2017 (22–25 April, Vienna, Austria).

Collaborative studies between hospitals in Europe and across the USA provided new insights into assessing susceptibility patterns and optimizing antimicrobial therapy in patients with infections caused by Gram-negative bacteria, including carbapenemase-producing Enterobacteriaceae (CPE) and *Pseudomonas aeruginosa*.

European Congress of Clinical Microbiology and Infectious Diseases 2017 (22–25 April, Vienna, Austria).

Collaborative studies between hospitals in Europe and across the USA provided new insights into assessing susceptibility patterns and optimizing antimicrobial therapy in patients with infections caused by Gram-negative bacteria, including carbapenemase-producing *Enterobacteriaceae* (CPE) and *Pseudomonas aeruginosa*.

## Simple mortality risk score guides when to use combination antibiotic therapy

A simple mortality risk score can be used to predict which patients with bloodstream infections caused by antibiotic-resistant CPE need combination antibiotic therapy and which patients need only one antibiotic, according to results from a large European study reported at the congress and published simultaneously in *Lancet Infectious Diseases* [1].

CPE are of growing concern because there are very few therapeutic options that are effective against these bacteria. Most CPE are resistant to all first-line anti-Gram-negative antibiotics. Several studies have suggested that combination therapy may be better than monotherapy but the optimal approach has previously been unclear.

The study retrospectively reviewed 437 patients with bloodstream infections due to CPE (most frequently *Klebsiella pneumoniae*) cared for in 26 hospitals across ten European countries. Before starting antimicrobial treatment, patients were assessed for their risk of death based on five variables giving their INCREMENT-CPE score: severe sepsis or shock at presentation (5 points), a Pitt bacteraemia score ≥6 (4 points), a Charlson comorbidity index score of ≥2 (3 points), a source of bloodstream infection other than the urinary or biliary tracts (3 points) and inappropriate empirical and early targeted therapy (2 points).

Use of combination antibiotic therapy was associated with reduced risk of death (44% reduction, hazard ratio 0.56, p = 0.02) compared with monotherapy only in patients at high risk of death with a score of 8–15 and not in those at low risk of death (scoring 0–7).

Contrary to present recommendations, combination therapy can be avoided in a substantial proportion of patients with bloodstream infections due to CPE, said study author Professor Jesús Rodríguez-Baño, from the University Hospital Virgen Macarena in Seville, Spain. He added that these patients can be identified using the INCREMENT-CPE score and if they are of low risk they can be treated with a single active antimicrobial. This will help to reduce the development of resistance to multiple antibiotics, as well as reducing side effects and costs of treatment, he concluded.

## Carbapenem nonsusceptibility rates twice as high in ICU as non-ICU settings

Rates of nonsusceptibility of Gram-negative bacteria to carbapenems are nearly twice as high in patients in intensive care unit (ICU) settings compared with those in non-ICU settings, reported a large US study [2].

The study analyzed an electronic research dataset of information on Gram-negative bacteria isolate tests from 342 US hospitals for over 1 year from January 2016 to December 2016. The hospitals reported data from nonduplicate Gram-negative isolates based on the first isolate of a species in each 30-day period, including *Escherichia coli*, *K. pneumoniae*, *Proteus mirabilis*, *Enterobacter aerogenes* and *Pseudomonas aeruginosa*. The isolates came from all sources, including urine, intra-abdominal, respiratory, skin and blood samples. *P. aeruginosa* isolates were classified as being carbapanem nonsusceptible if they showed intermediate response or were resistant to imipenem or meropenem. *Enterobacteriaceae* spp. showing intermediate response or resistance to both of these carbapenems plus ertapenem were classified as nonsusceptible.

Results showed that the carbapenem nonsusceptibility rate was nearly twice as high in the ICU as in non-ICU settings. Nearly one in ten Gram-negative isolates were carbapenem nonsusceptible in ICU settings (9.6%, 2759/28 619) compared with 5.1% (5653/111 607) in isolates from non-ICU settings. This gave an odds ratio for carbapanem nonsusceptibility in ICU compared with non-ICU settings of 1.69 (95% CI: 1.53–1.86; p < 0.0001).

Further analysis revealed that carbapenem nonsusceptibility rates in the ICU were highest in *P. aeruginosa* isolates (30.0%), which accounted for more than two-thirds (69.7%) of all ICU nonsusceptible isolates. Rates were also high among *K. pneumoniae* (6.1%), which accounted for 11.9% of carbapenem nonsusceptible Gram-negative isolates from ICUs.

The study authors, led by Eilish McCann (Merck & Co, NJ, USA), said the findings highlight the importance of understanding the variability in carbapenem susceptibility among isolates from different treatment settings in hospitals to inform appropriate prescribing of antibiotics.

## Identify patients with intra-abdominal infections due to Gram-negative pathogens to plan management

It is essential to identify patients with intra-abdominal infections (IAIs) at risk for infection with Gram-negative pathogens and treat with broad-spectrum antimicrobials, suggested Andrew Shorr (MedStar Washington Hospital Center, WA, USA), during a symposium on the challenge of resistant infections due to *Pseudomonas* and ESBL-producing Enterobacteriaceae.

Drawing on new guidelines on IAIs published recently by the Surgical Infections Society [3], Shorr suggested that clinicians should stratify all patients with IAIs as being at lower or higher risk for poor outcomes including treatment failure or death. He explained that risk factors for poor outcomes include severity of illness, risk for mortality, extent of infection and presence of septic shock. Patients should also be stratified according to whether they have a community-acquired or healthcare-associated infection as part of planning their management, which should include identifying patients known to have been colonized or infected previously with a resistant pathogen.

In terms of treatment, Shorr explained that the new guidelines recommend using antimicrobial regimens with activity against the typical Gram-negative Enterobacteriaceae, Gram-positive cocci and obligate anaerobes often involved in IAIs. Higher risk patients with community-acquired or hospital-acquired IAIs should be treated with broader spectrum antimicrobial agents to ensure coverage of less common Gram-negative pathogens that can potentially be involved in these infections.

He noted the importance of identifying patients at risk for infection with resistant Gram-negative pathogens. Risk factors include:

- Substantial previous broad-spectrum antimicrobial therapy;
- Prolonged hospitalizations;
- Multiple invasive interventions;
- Previous infection with a resistant Gram-negative pathogen.

Use of a broad-spectrum carbapenem, ceftolozane-tazobactam or ceftazidime-avibactam should be considered for empiric therapy of patients at risk for infection with ESBL-producing Enterobacteriaceae, he explained.

Reviewing the risk factors for resistance in IAIs from epidemiology studies and antimicrobial resistance reports, Shorr reported that they include: general prevalence (e.g., pretest probability), based on national figures, and data from the hospital and unit; known colonization; severity of illness; nosocomial onset; and re-operation. Data have shown that resistance occurs in a significantly higher percentage of isolates from patients in ICU compared with those cared for in non-ICU settings. For healthcare-associated infections, the particular risk factors for resistance in IAIs include: recent hospitalization; prior antibiotic exposure; nursing home/long term care; wound care; hemodialysis; and chemotherapy/immunosuppression.

Shorr reported recent data from the Study for Monitoring Antimicrobial Resistance Trends program in the US, which evaluated the *in vitro* activity of several antimicrobial agents recommended for treatment of IAIs and compared profiles of isolates of Gram-negative bacilli collected from patients hospitalized for IAIs in ICUs with those from non-ICUs. Results for 2010–2012 showed that 29.5% of ICU patients were infected with Gram-negative bacilli that were resistant to at least two antibacterial classes. A total of 28.5% had *Enterobacteriaceae* resistance to at least two antibacterial classes, and 29.6% had infections involving *P. aeruginosa* resistance to at least two classes. Intensive care isolates were more likely to be multidrug resistant than non-ICU isolates, although there was no difference in ESBL production rates between the two patient groups [4].

Appropriate initial therapy has a major impact on patient mortality, Shorr told the meeting. He illustrated this with findings from a study of just over 2000 patients with septic shock [5]. Results showed that administration of an antimicrobial agent for isolated or suspected pathogens within the first hour of documented hypotension was associated with an in-hospital survival rate of 79.9%. Each hour of delay in starting antimicrobial therapy was associated with 12% increase in mortality. By the second hour after persistent hypotension onset, in-hospital mortality was 67% higher (odds ratio [OR]: 1.67, 95% CI: 1.12–2.48) than starting therapy within the first hour and this increased to more than ninety times higher (OR: 92.54) with delays over 36 h. Multivariate analysis revealed that timing to initiating effective antimicrobial therapy was the single strongest predictor of outcome, he noted.

A further study that retrospectively analyzed the impact of appropriate antimicrobial therapy on mortality in 1250 patients with septic shock showed that the number needed to treat to prevent one patient death was only five. Shorr compared this to a number needed to treat of around 1000 for passenger air bags in cars. Use of inappropriate antibiotics was associated with a 3.4-fold increase in risk for death. The results suggested that improved targeted of empiric antimicrobials for multidrug-resistant bacteria, methicillin-resistant *S. aureus* and *Candida* species would have the greatest impact in reducing mortality from inappropriate antimicrobial treatment in patients with severe sepsis and septic shock [6].

In conclusion, Shorr warned that resistance among Gram-negative rods is increasing, with effects at a global level and across multiple pathogens. He cautioned that although predictive factors can be used to assess risk of resistance they have limitations and can be affected by type of infection, pathogen, severity of illness and country. Resistance drives inappropriate therapy, he cautioned, adding that the key measure to reduce this serious and growing threat is for clinicians to be aware of local resistance patterns and to balance this information against the risk of inappropriate therapy. He noted that newer options and strategies are now becoming available to meet the challenge of IAIs.

## Emerging data with fifth-generation cephalosporins

Tracing the real-world use of fifth generation cephalosporins, Matteo Bassetti (Santa Maria Misericordia University Hospital, Udine, Italy) reported that both ceptobiprole and ceftaroline fosamil have broad-spectrum activity. Considering the ideal characteristics of an anti-staphylococcal agent, he suggested that it should be equally active against multiresistant *S. aureus*, hetero-resistant and vancomycin-intermediate *S. aureus* (hVISA) and methicillin-resistant *S. aureus*. It should have a rapid effect that is bactericidal, good tissue penetration in lung, bone, skin and cerebrospinal fluid and a good safety profile, he told at a symposium held during the conference [7].

Reviewing the current challenges in treating complicated skin and soft tissue infection (cSSTI) with vancomycin and teicoplanin, Professor Bassetti pointed out that vancomycin is only moderately bactericidal and the susceptibility of *S. aureus* may be compromised. Hetero-resistant and vancomycin-intermediate *S. aureus* are clinically associated with vancomycin failure and increased vancomycin minimum inhibitory concentrations (MICs; ≥1.5 μg/ml) are associated with significantly increased mortality. In addition, vancomycin ‘MIC creep’ in methicillin-resistant *S. aureus* (MRSA) isolates may be associated with cross-resistance to daptomycin. He warned that vancomycin is less effective against MRSA than β-lactams. Aggressive vancomycin dosing regimens are associated with unacceptably high microbiological failure rates and noted that target attainment at vancomycin MICs of 2 mg/l is not possible. High-dose vancomycin has been confirmed to be nephrotoxic.

Looking at real world data on the use of antibiotics in skin infections and community-acquired pneumonia (CAP), Professor Bassetti warned that patients frequently have comorbidities underlining the need for antibacterial agents with good safety profiles. A recent study showed that 40.7% of patients with skin infections had diabetes and 29.3% had peripheral vascular disease. Nearly one in ten patients (9.3% had congestive heart failure and 8.7% had chronic renal failure or other renal disease [8]. High rates of diabetes (17.6%) and peripheral vascular disease (13.4%) were also seen in the CANVAS 1 and 2 trials comparing ceftaroline fosamil to vancomycin plus aztreonam in patients with complicated skin and skin-struture infections [9].

CANVAS 1 randomized adult patients with cSSTI and systemic inflammation or comorbidities to intravenous ceftaroline (600 mg every 8 h) or vancomycin (15 mg/kg every 12 h) plus aztreonam (1 g every 8 h) for 5–14 days. After the main study was completed an MRSA-focused extension study enrolled 802 patients randomized to ceftaroline or vancomycin plus aztreonam. Clinical cure rates at the test of cure timepoint (8 to 15 days after the final dose) were noninferior with ceftaroline (78.3 vs 79.2% for the modified intention to treat population) and the frequency of adverse events was similar between the two treatment groups [10].

The CAPTURE study in patients with community acquired pneumonia (344 patients) or SSTI (985 patients) showed similar clinical success rates with ceftaroline fosamil across patients with varying degrees of renal insufficiency [11]. The clinical success rate was 91% in patients treated for SSTIs with normal renal function or mild renal insufficiency, 88% in those with moderate renal insufficiency and 89% in patients with severe renal insufficiency. Further results showed that ceftaroline achieved high rates of clinical success when used as first- or second-line therapy to treat CAP and SSTIs [12].

Considering the broader treatment of CAP, Professor Bassetti explained that patient risk factors associated with a poor prognosis include older age (>65 years), diabetes, renal disease, congestive heart failure and malnutrition [13]. He advised that selection of optimal antimicrobial regimens is best achieved by stratifying patients according to:

- Medical comorbidities that predict the likelihood of clinical failure and/or mortality;
- Specific risk of resistance [14].

The CAPTURE study showed that ceftaroline fosamil is a viable option for treating CAP in the elderly, with similar clinical success in patients aged 65 years and older (81%) to patients younger than 65 (82%) [15].

Summing up, Professor Bassetti warned that glycopeptides, such as vancomycin and teicoplanin, present problems in the treatment of staphylococcal infections. He explained that cefataroline is the first available β-lactam to have activity against MRSA and other common Gram-positive and Gram-negative pathogens causing complicated SSTIs and respiratory infections. Data from the PREMIUM study showed that ceftaroline has *in vitro* activity at the breakpoint concentration against the majority of isolates collected from patients across Europe, including 94.1% of MRSA [16]. Bactericidal activity occurs with earlier response at day 3–4 in cSSTI and CAP and higher cure rates have been seen than with ceftatriaxone in *S. pneumoniae* pneumonia [17].

## Real world data show antibacterial activity of ceftozolane/tazobactam against *P. aeruginosa* isolates in bloodstream infections & pneumonia across Europe

Ceftozolane/tazobactam demonstrated potent activity against bloodstream infections and pneumonia *P. aeruginosa* isolates from patients in hospitals across Europe, reported a large study [18].

The study analyzed 1945 *P. aeruginosa* isolates collected from patients with bloodstream infections (503 isolates) and pneumonia (1442 isolates) in 42 European hospitals between 2014 and 2016. Isolates were tested for susceptibility to ceftozolane/tazobactam at a central monitoring laboratory. Other antibiotics were also tested, including amikacin, cefepime, ceftazidime, colistin, levofloxacin, meropenem and piperacillin/tazobactam.

Results reported at ECCMID 2017 showed that 91.5% of *P. aeruginosa* isolates from patients with bloodstream infections were susceptible to ceftozolane/tazobactam at ≤4 mg/l. For the other antibiotics tested, 88.3% of bloodstream isolates were susceptible to amikacin, 83.5% to cefepime, 78.9% to ceftazadime, 100% to colistin, 69.0% to levofloxacin, 78.5% to meropenem and 77.8% to piperacillin/tazobactam.

For *P. aeruginosa* isolates from patients with pneumonia, ceftozolane/tazobactam inhibited growth of 90.1% at ≤4 mg/l. A total of 82.2% of isolates were susceptible to amikacin, 77.7% to cefepime, 72.9% to ceftazadime, 99.9% to colistin, 59.2% to levofloxacin, 69.3% to meropenem and 70.1% to piperacillin/tazobactam.

Analyzing resistance patterns showed that pneumonia isolates were more resistant than isolates from patients with bloodstream infections and had a higher percentage of resistant phenotypes tested in the study. Nearly one in three (29.6%) pneumonia *P. aeruginosa* isolates were multidrug resistant compared with 20.7% of bloodstream infection isolates. A total of 22.6% of pneumonia isolates were extensively drug resistant, while this resistance phenotype occurred in 15.5% of *P. aeruginosa* isolates from bloodstream infections.

The researchers concluded that ceftozolane/tazobactam showed highly potent activity against bloodstream infection and pneumonia *P. aeruginosa* isolates. They noted that ceftozolane/tazobactam was more active than all comparator antibiotics tested in the study apart from colistin.

## Relebactam restores activity of imipenem in *P. aeruginosa* isolates nonsusceptible to carbapenem

The investigational non-β-lactam β-lactamase inhibitor relebactam restored *in vitro* activity of imipenem against around three-quarters of isolates of *P. aeruginosa* that were nonsusceptible to carbapenem treatment collected as part of the global Study for Monitoring Antimicrobial Resistance Trends surveillance program, latest data reported at the congress [19].

The study collected a total of 5491 isolates of *P. aeruginosa* from 154 medical centers in 51 countries. Each center collected consecutive, nonduplicate Gram-negative isolates from patients with respiratory tract, intra-abdominal, urinary tract infections and from those with infections of unknown source. Isolates were tested for antimicrobial susceptibility and results showed that just under a third (31.3%) of *P. aeruginosa* isolates from respiratory tract infections were nonsusceptible to imipenem. Nonsusceptibiity to imipenem was seen in 24.2% of intra-abdominal isolates, 25.5% of urinary tract isolates and 23.5% of isolates from unknown infection sources.

The percentage of *P. aeruginosa* isolates that were nonsusceptible to imipenem ranged from 14.1% in the South Pacific to 36.4% in Latin America. The addition of relebactam restored susceptibility to imipenem (minimum inhibitory concentration ≤2 mg/l) to between 75.3 and 77.8% of imipenem-nonsusceptible isolates collected in Europe, North America, Asia, South America, Asia, South Pacific and the Middle East regions, for overall susceptibilities of 91.6–96.8%.

The study authors concluded that further development of imipenem/relebactam/cilastatin could provide a valuable therapeutic option for treating infections caused by resistant isolates of *P. aeruginosa.*
